# Advanced Life Support vs. Basic Life Support for Patients With Trauma in Prehospital Settings: A Systematic Review and Meta-Analysis

**DOI:** 10.3389/fmed.2021.660367

**Published:** 2021-03-26

**Authors:** Yutaka Kondo, Tatsuma Fukuda, Ryo Uchimido, Masahiro Kashiura, Soichiro Kato, Hiroshi Sekiguchi, Yoshito Zamami, Toru Hifumi, Kei Hayashida

**Affiliations:** ^1^Department of Emergency and Critical Care Medicine, Juntendo University Urayasu Hospital, Chiba, Japan; ^2^Department of Emergency and Critical Care Medicine, University of the Ryukyus, Okinawa, Japan; ^3^Department of Intensive Care Medicine, Tokyo Medical and Dental University, Tokyo, Japan; ^4^Department of Emergency and Critical Care Medicine, Jichi Medical University Saitama Medical Center, Saitama, Japan; ^5^Department of Trauma and Critical Care Medicine, Kyorin University School of Medicine, Tokyo, Japan; ^6^Department of Clinical Pharmacology and Therapeutics, Institute of Biomedical Sciences, Tokushima University Graduate School, Tokushima, Japan; ^7^Department of Emergency and Critical Care Medicine, St. Lukes International Hospital, Tokyo, Japan; ^8^Department of Emergency Medicine, Feinstein Institutes for Medical Research, Northwell Health, New York, NY, United States

**Keywords:** first aid, emergency medical services, resuscitation, mortality, injury

## Abstract

**Background:** Advanced Life Support (ALS) is regarded to be associated with improved survival in pre-hospital trauma care when compared to Basic Life Support (BLS) irrespective of lack of evidence. The aim of this study is to ascertain ALS improves survival for trauma in prehospital settings when compared to BLS.

**Methods:** We searched PubMed, EMBASE, and the Cochrane Central Register of Controlled Trials for published controlled trials (CTs), and observational studies that were published until Aug 2017. The population of interest were adults (>18 years old) trauma patients who were transported by ground transportation and required resuscitation in prehospital settings. We compared outcomes between the ALS and BLS groups. The primary outcome was in-hospital mortality and secondary outcomes were neurological outcome and time spent on scene.

**Results:** We identified 2,502 studies from various databases and 10 studies were included in the analysis (two CTs, and eight observational studies). The outcomes were not statistically significant between the ALS and BLS groups (pooled OR 1.14; 95% CI 0.95 to 1.36 for mortality, pooled OR 1.12; 95% CI 0.88 to 1.42 for good neurological outcomes, pooled mean difference −0.96; 95% CI−6.64 to 4.72 for on-scene time) in CTs. In observational studies, ALS prolonged on-scene time and increased mortality (pooled OR 1.56; 95% CI: 1.31 to 1.86 for mortality, and pooled mean difference, 1.26; 95% CI: 0.07 to 2.45 for on-scene time).

**Conclusions:** In prehospital settings, the present study showed no advantages of ALS on the outcomes in patients with trauma compared to BLS.

## Introduction

Advanced Life Support (ALS) is widely accepted as the standard of prehospital care in patients with cardiac arrest caused by internal diseases (1–3). ALS procedure includes invasive interventions, such as endotracheal intubation for airway management, and intravenous catheters for drug and fluid delivery. ALS is also used to resuscitate trauma patients in prehospital settings.

On the contrary, an observational study using two large registry data sets reported that prehospital ALS procedures in patients with trauma were not associated with increased survival rate (4). Furthermore, other studies reported that prehospital ALS increased the spending time on the scene and thus delayed definitive in-hospital care (5, 6). Rapid transportation to the hospital is required as in-hospital surgery is typically needed to improve the prognosis of trauma patients.

Some researchers argue that basic life support (BLS) is more beneficial for trauma because of rapid transportation (5). Prehospital BLS consists of non-invasive interventions that are easy to perform, require little added on-scene time, and can often be performed *en route* to a medical facility by minimally trained emergency medical staff. Thus, the benefits of prehospital ALS on trauma have not been clearly established yet (7–9).

The aim of the present study is to clarify if ALS improves survival in patients with trauma in prehospital settings when compared to BLS by conducting a systematic review and meta-analysis.

## Methods and Analysis

### Ethics and Approval

This systematic review and meta-analysis protocol has been registered in PROSPERO, an International Prospective Register of Systematic Reviews at the National Institute for Health Research and Center for Reviews and Dissemination (CRD) at the University of York (http://www.crd.york.ac.uk/PROSPERO/; registration no. CRD42017054389) (10). The protocol also has already been published (11).

The systematic review and meta-analysis was reported in accordance with Preferred Reporting Items for Systematic Reviews and Meta-Analyses (PRISMA) (12, 13) and Meta-analysis Of Observational Studies in Epidemiology (MOOSE) guidelines and does not require ethical approval (14).

### Search Strategies

Database searches were conducted in MEDLINE (*via* PubMed), EMBASE, and the Cochrane Central Register of Controlled Trials (CENTRAL) to retrieve relevant articles for the literature review. We searched for full-text controlled trials (CTs) [included controlled before-and-after studies (CBAs), randomized controlled trials (RCTs)], and observational studies in humans that were published until Aug 2017. We used a combination of key terms and established a full search strategy (Supplementary Material 1).

### Study Selection and Inclusion Criteria

CTs, CBAs, RCTs, and observational studies were included. We defined CTs, CBAs, and RCTs, as the CTs design group and prospective or retrospective observational study as the observational study (OS) design group.

Our study population of interest was adults (>18 years old) trauma patients who were transported by ground and required resuscitation in prehospital settings. We did not restrict our analysis by country and included all severities and types of trauma. Conference abstracts, studies in animals, and those that only include trauma patients transported by helicopter were excluded. We only included studies which were written in English or Japanese.

The interventions of interest are ALS and BLS. The ALS group was defined as having undergone one or more of the following intervention components: (1) tracheal intubation, (2) needle tracheostomy, and administration of (3) intravenous (IV) fluids, (4) epinephrine, or (5) other IV drugs (e.g., amiodarone, lidocaine, or magnesium). The BLS group was defined as not having undergone any of the above ALS procedures, only BLS was instituted (chest compression, mouth-to-mouth breathing, bag valve mask ventilation, and automated external defibrillator).

We compared the outcomes between the ALS and BLS groups. Primary outcome was in-hospital mortality and secondary outcomes were neurological outcome and time spent on scene (minutes).

### Assessment of Risk of Bias

To assess the quality of the included studies, we adapted the Cochrane risk of bias tool for CTs design (15). Each study was assessed for: (1) random sequence generation (selection bias), (2) allocation concealment (selection bias), (3) blinding of participants and personnel (performance bias), (4) blinding of related outcomes assessment (detection bias), (5) incomplete outcome data (attrition bias), (6) selective reporting (reporting bias), and (7) other bias. Studies were categorized as having a low, unclear, or high risk of bias in each domain. The risk of bias for each element was considered “high” when bias was present and likely to affect outcomes and “low” when bias was not present or present but unlikely to affect outcomes (16). For OS design, we applied the Risk of Bias Assessment Tool for Non-randomized Studies (RoBANS) to assess the risk of bias of observational studies, which is compatible with the Cochrane risk-of-bias tool (17). Two independent reviewers (YK and HS) chosen by the authors performed the risk of bias assessment. Disagreements were resolved through discussion.

### Data Extraction and Management

**Figure 1:**
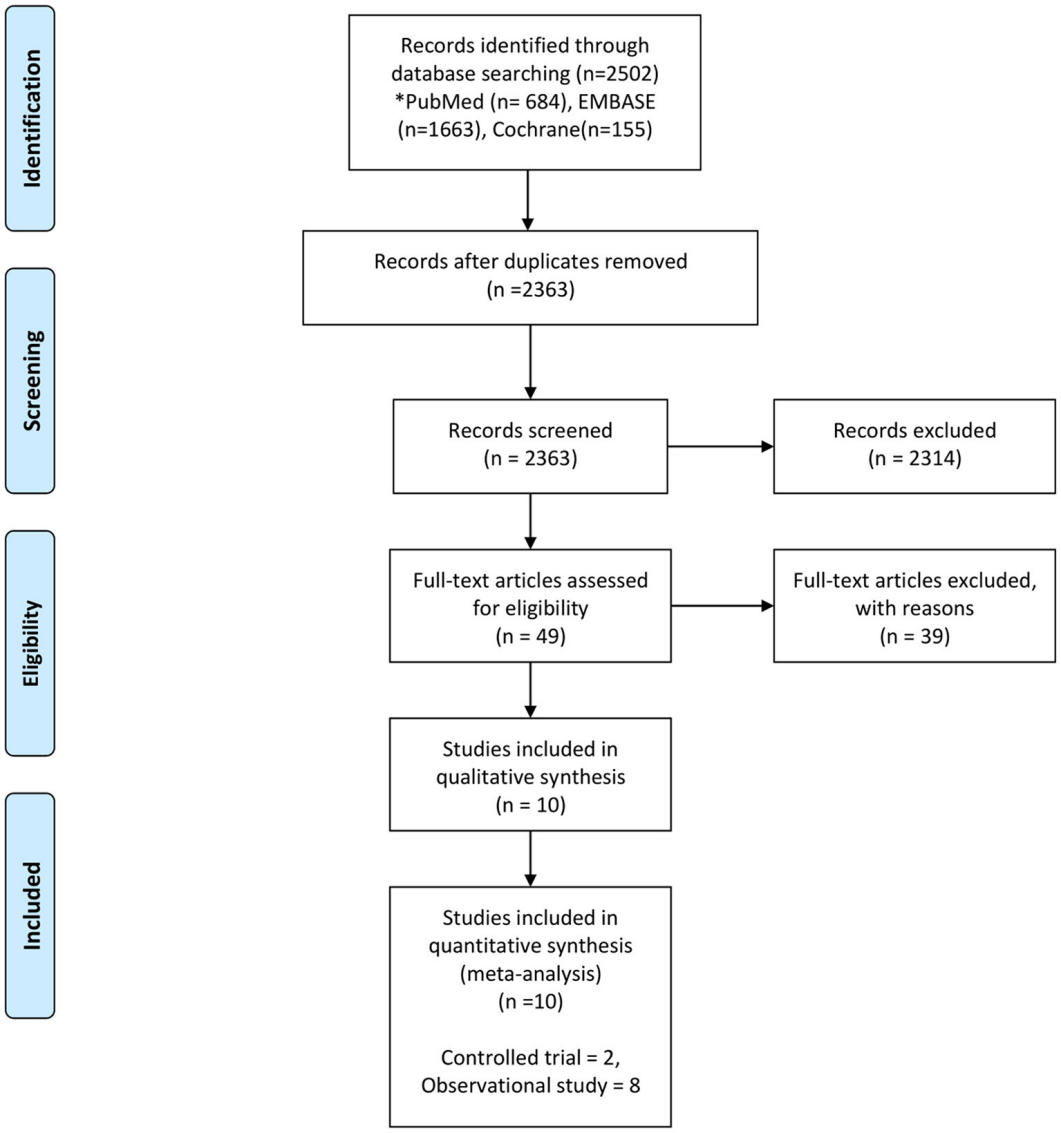
Flow chart of the study selection process From Moher et al. (13).

The following data were extracted: author(s), title, journal name, year of publication, website (URL), and abstract. After removal of duplicates, two independent reviewers (MK and SK) screened the abstracts and titles of the studies and subsequently reviewed the full-text articles for inclusion using an electronic screening form (Covidence web platform: http://www.COVIDENCE.org). Disagreements were reconsidered and discussed until a consensus was reached. The full-text of the articles included in the final selection was independently reviewed by other two reviewers (TF and UR). Disagreements were solved by a third reviewer (YK). The flow diagram of our study, has been adapted from the PRISMA statement (2009) (13), (Figure 1).

### Rating the Quality of Evidence Using the GRADE Approach

We used the Grading of Recommendations Assessment, Development and Evaluation (GRADE) tool to rate the quality of the evidence on the effect of ALS and BLS on important outcomes in trauma patients (18–21). The quality of evidence was assessed for each outcome and categorized as high, moderate, low, or very low using the GRADE pro Guideline Development Tool.

### Statistical Analysis

We performed a meta-analysis because one or more data were available according to the “Cochrane Handbook for Systematic Reviews of Interventions” and the PRISMA guidelines. Results were summarized using a random effects model to facilitate pooling of estimates of the treatment effects. Odds ratios (OR) and 95% confidence intervals (CI) were used for dichotomous outcomes and mean differences and 95% CIs for continuous outcomes. Heterogeneity between trials for each outcome was evaluated using the *I*^2^ statistic for quantifying inconsistency (22). We considered heterogeneity as being significant if the reason for heterogeneity could not be explained, and if *I*^2^ was 50% or greater.

Regarding assessment of reporting bias, we investigated the potential for publication bias using a funnel plot. Estimates were pooled using a random effects model. The meta-analysis was performed based on all published data and data made available to us (16).

All analyses were performed by using the Review Manager software (RevMan 5.3, Copenhagen, Denmark: The Nordic Cochrane Centre, the Cochrane Collaboration 2014).

## Results

We identified 2,502 studies from the electronic databases. We eliminated 139 duplicates and excluded 2,314 studies because their design did not fit. Finally, we retained 16 studies for review of the full lengths reports and included 10 studies (5, 6, 23–30) in the final analysis (Figure 1).

### Study Characteristics

**Figure 2:**
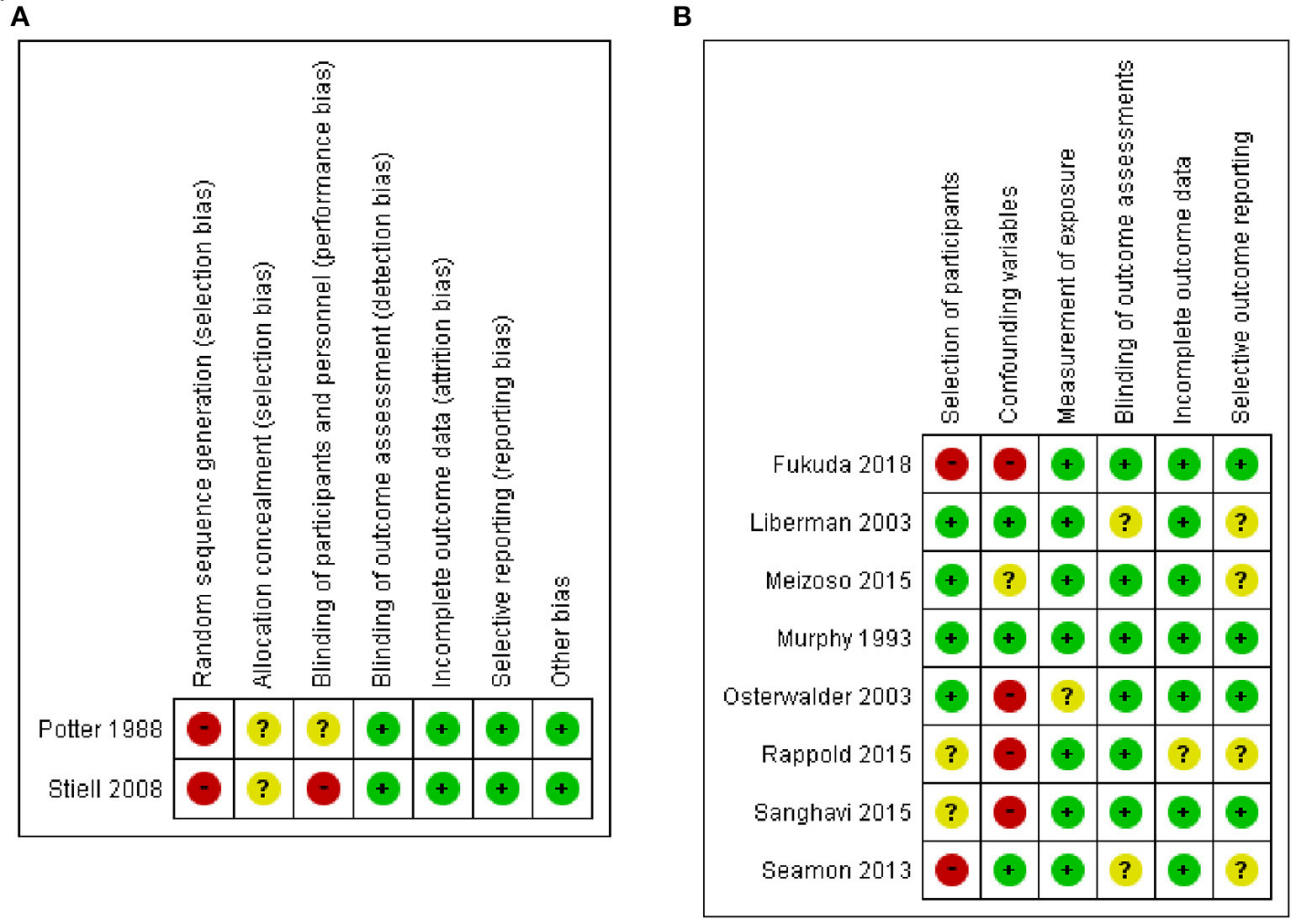
Risk of bias summary in included studies: **(A)** based on the criteria recommended by the Cochrane Collaboration for RCTs, and **(B)** based on the Risk of Bias Assessment Tool for Nonrandomized Studies for observational studies.

The 10 studies (5, 6, 23–30) included 105,451 patients (two studies for the CTs design group, and eight studies for the OS design group) (Table 1). In the CTs design group, 1,966 were assigned to the ALS group and 1,962 to the BLS group. In the OS design group, 54,982 were assigned to the ALS group and 42,080 to the BLS group. Five studies (5, 6, 24, 28, 29) took place in United States, two in Canada (27, 31), and one each in Australia (23), Switzerland (26), and Japan (30). Four studies were conducted prospectively (one CBAs, one CTs, and two observational studies), and the others were retrospective. The risk of bias was evaluated for each study in the CTs design group and is shown in the risk of bias summary (Figure 2). Because only one CBAs and one CTs were included as the CTs group, random sequence generation could not be performed and was rated high risk selection bias in these two studies. The risk of bias assessment of the observational studies was done using RoBANS (Figure 2B).

**Table 1:** Baseline characteristics of eligible studies.

**No**.	**References**	**Country**	**Design**	**Number of study participants**	**Type of trauma**	**Body region**	**ISS (mean or median)**	**Performed by**		**Procedures**	
								**BLS**	**ALS**	**BLS**	**ALS**
1	Potter et al. (23)	Australia	Controlled Trial	1,061	Blunt and Penetrating	Head, Torso and Extremity	37	Physician and Paramedics	Physician and Paramedics	All BLS procedures	All ALS procedures
2	Murphy et al. (24)	US	Retrospective Cohort	2,394	Blunt and Penetrating	Head, Torso and Extremity with multiple injuries	17	Not described	Not described	All BLS procedures	All ALS procedures
3	Liberman et al. (25)	Canada	Prospective Cohort	9405	Blunt and Penetrating	Head, Torso and Extremity	26	Paramedics	Physician and Paramedics	All BLS procedures	All ALS procedures
4	Osterwalder (26)	Switzerland	Prospective Cohort	196	Blunt	Head, Torso and Extremity	24	Physician and Paramedics	Physician and Paramedics	All BLS procedures	All ALS procedures
5	Steil (27)	Canada	Before-after controlled trial	2,867	Blunt, Penetrating and Burn	Head, Torso and Extremity	24 for BLS, 22 for ALS	Paramedics	Paramedics	All BLS procedures	All ALS procedures
6	Seamon et al. (6)	US	Prospective Cohort	236	Penetrating	Head, Torso and Extremity	20.8	Paramedics	Paramedics	All BLS procedures	All ALS procedures
7	Meizono (28)	US	Retrospective Cohort	3,733 (122, after adjustment)	Blunt, Penetrating and Burn	Not described	5	Not described	Not described	No Procedures of ALS group	^*^ALS procedures
8	Sanghavi et al. (29)	US	Retrospective Cohort	79,687	Not described	Not described	New ISS was used.	Not described	Not described	All BLS procedures	All ALS procedures
9	Rappold et al. (5)	US	Retrospective Cohort	1,490	Penetrating	Not described	13 for ALS, 10 for BLS	Paramedics	Paramedics	All BLS procedures	All ALS procedures
10	Fukuda et al. (30)	Japan	Retrospective Cohort	4,382	Blunt and Penetrating	Head, Torso and Extremity	Unknown	Physician and Paramedics	Physician and Paramedics	All BLS procedures	All ALS procedures

*ISS, injury severity score; BLS, basic life support; ALS, advanced life support; US, United States*.

* *Needle decompression, tourniquet use, cricothyroidotomy, or ACLS procedures other than intravenous fluid*.

### Outcomes

**Figure 3:**
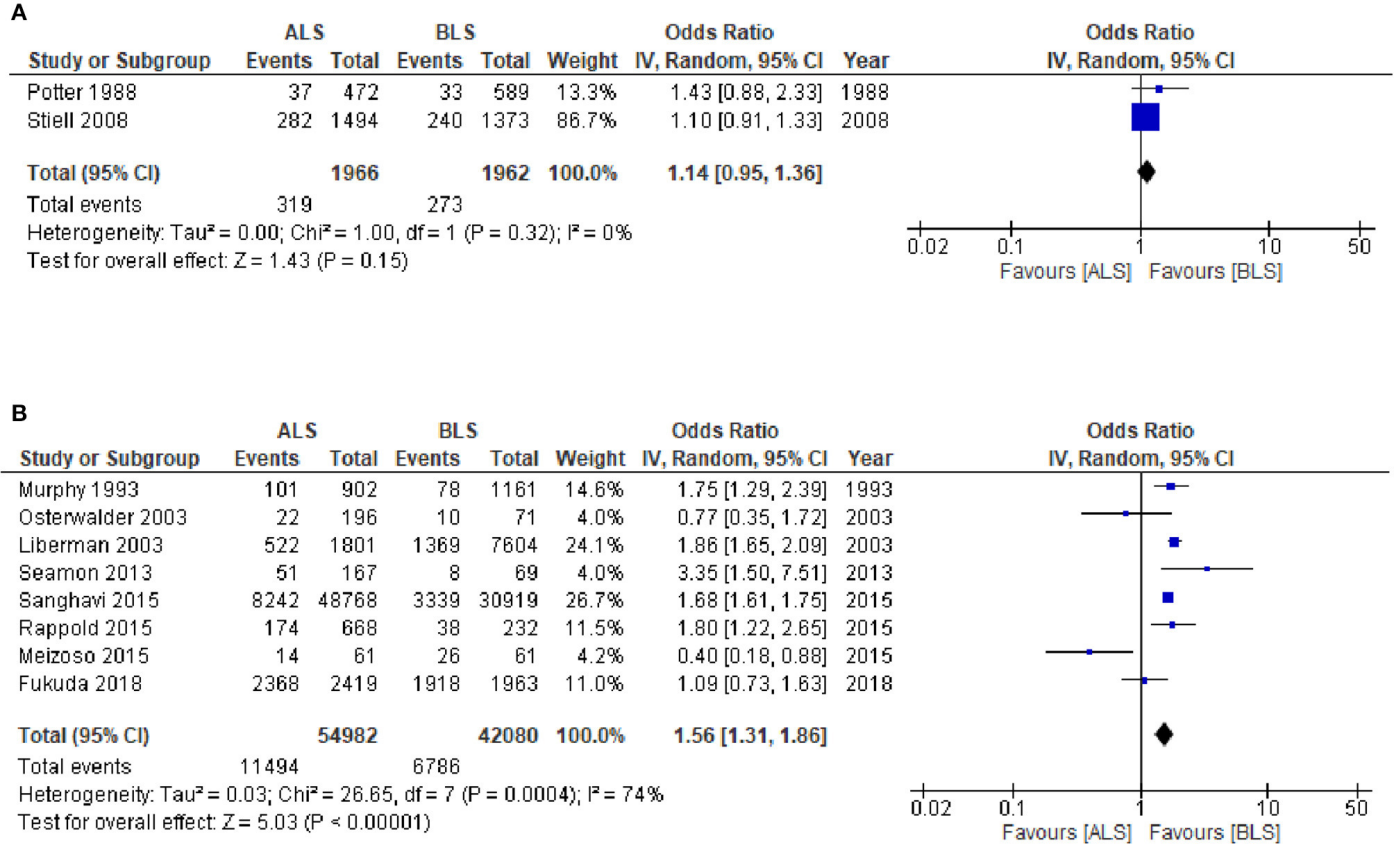
Forest plot of the comparison: ALS vs. BLS for in-hospital mortality **(A)** Controlled trials; **(B)** Observational studies. ALS, advanced life support, BLS, basic life support, IV, inverse variance weighted method, CI, confidence interval.

**Figure 4:**
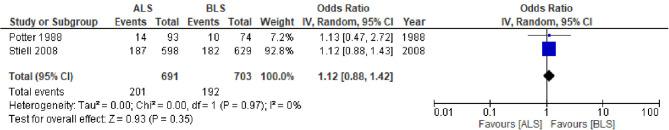
Forest plot of the comparison: ALS vs. BLS for neurological outcomes. ALS, advanced life support; BLS, basic life support; IV, inverse variance weighted method; CI, confidence interval.

**Figure 5:**
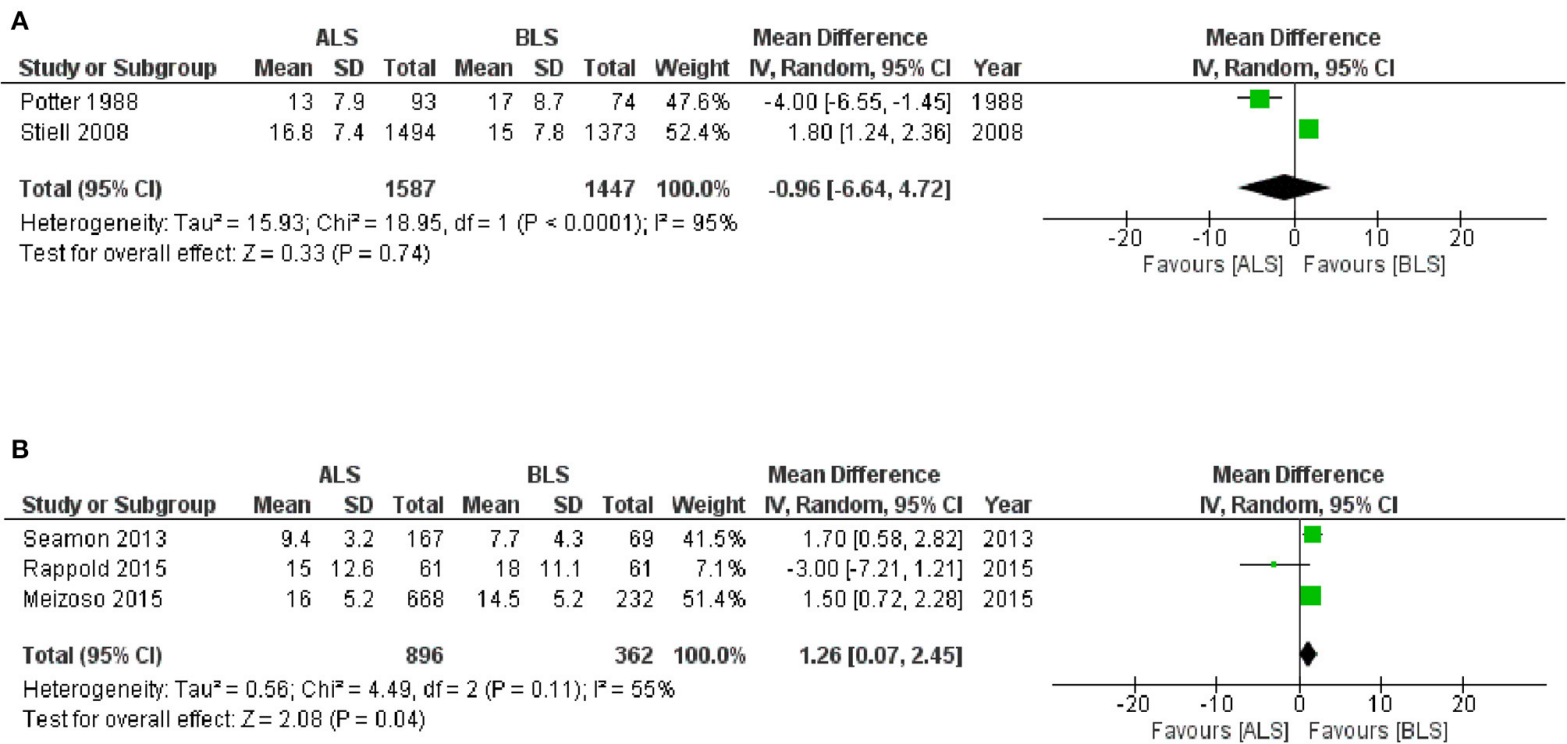
Forest plot of the comparison: ALS vs. BLS for on-scene spending time **(A)** controlled trials; **(B)** observational studies. The unit of number in mean is minutes. ALS, advanced life support; BLS, basic life support, IV, Inverse variance weighted method; CI, confidence interval.

The CTs design group with 3,928 patients (two studies) reported in-hospital mortality as a primary outcome with 1,966 patients in the ALS group and 1,962 patients in the BLS group (Figure 3A). Of these, 319 patients (16.2%) died in the ALS group and 273 patients (13.9%) died in the BLS group. The pooled OR of mortality was not statistically significant (OR 1.14; 95% CI 0.95 to 1.36) (Figure 3A). When comparing neurological outcomes and time spent on scene, there were no significant differences between the ALS and BLS groups. The pooled OR was 1.12 (95% CI: 0.88 to 1.42) for neurological outcomes, and −0.96 (95% CI: −6.64 to 4.72) for time spent on scene (Figures 4, 5A).

The OS design group which had 97,062 patients (eight studies) reported in-hospital mortality as a primary outcome and of these, 54,982 patients belonged to the ALS group and 42,080 patients belonged to the BLS group (Figure 3B). Of these, 11,494 patients (20.9%) died in the ALS group and 6786 patients (16.1%) died in the BLS group. The pooled OR of mortality was statistically significant in favor of BLS (pooled OR 1.56; 95% CI: 1.31 to 1.86) (Figure 3B). None of the studies included information regarding neurological outcomes. Time spent on scene was significantly prolonged in the ALS group (pooled mean difference, 1.26; 95% CI: 0.07 to 2.45) (Figure 5B).

### Heterogeneity

No statistically significant heterogeneity in short-term mortality was observed between the ALS and the BLS groups in the CTs group (I^2^ = 0%; χ^2^ = 1.00; *p* = 0.32) whereas the OS design groups showed statistical heterogeneity (I^2^ = 74.0%; χ^2^ = 26.65; *p* ≤ 0.001). No statistically significant heterogeneity in neurological outcomes was observed between the ALS and the BLS groups in the CTs design group (*I*^2^ = 0%; χ^2^ = 0.00; *p* = 0.97). A statistical heterogeneity was observed in time spent on scene (minutes) in the CTs design group (*I*^2^ = 95.0%; χ^2^ = 18.95; *p* ≤ 0.001) and the OS group (*I*^2^ = 55.0%; χ^2^ = 4.49; *p* = 0.11).

### Publication Biases, and Quality of Evidence

We tested for the presence of publication biases for the primary outcome. A visual inspection of the funnel plots suggested no existence of publication biases in the in-hospital mortality CTs design group whereas the OS design group showed publication biases (Supplementary Material 2).

The quality of evidence was rated as moderate due to the high risk of biases for the effect of ALS on the in-hospital mortality compared with BLS in the CTs design groups. The grade for in-hospital mortality in the OS design group was rated very low, due to an inconsistency, which the Cochrane chi-square test revealed to be a significant heterogeneity, and due to a publication bias. The quality of evidence was rated as moderate for the effect of ALS on neurological outcome, compared with BLS. The quality of evidence was rated as moderate due to high risk of biases for the effect of ALS on time spent on scene compared with BLS in the CTs design groups and the grade in the OS design group was rated low (Table 2).

**Table 2:** Summary of finding table.

**Outcomes**	Anticipated absolute effects^*^ **(95% CI)**		**Relative effect (95% CI)**	**No. of participants (studies)**	**Certainty of the evidence (GRADE)**
	**Risk with BLS**	**Risk with ALS**			
Mortality (CTs)	139 per 1,000	156 per 1,000 (133 to 180)	OR 1.14 (0.95 to 1.36)	3,928 (2 studies)	⊕ ⊕ ⊕ ○ Moderate
Mortality (OS)	161 per 1,000	231 per 1,000 (201 to 263)	OR 1.56 (1.31 to 1.86)	97,062 (8 studies)	⊕ ○○ ○ Very low
Disability of CNS (CTs)	273 per 1,000	296 per 1,000 (248 to 348)	OR 1.12 (0.88 to 1.42)	1,394 (2 studies)	⊕ ⊕ ○ ○ LOW
On-scene time (CTs)	The mean total time on scene was 0	MD 0.96 lower (6.64 lower to 4.72 higher)	-	3,034 (2 studies)	⊕ ⊕ ⊕ ○ Moderate
On-scene time (OS)	The mean on-scene time was 0	MD 1.26 higher (0.07 higher to 2.45 higher)	-	1,258 (3 studies)	⊕ ⊕ ○ ○ LOW

* *The risk in the ALS group (and its 95% confidence interval) is based on the assumed risk in the BLS group and the relative effect of the ALS (and its 95% CI)*.

*CI, confidence interval; BLS, basic life support; ALS, advanced life support; GRADE, The Grading of Recommendations Assessment, Development and Evaluation, RCT, randomized control trial; OS, observational study; OR, odds ratio; CNS, central nerve system; MD, mean difference*.

## Discussion

In this systematic review, we have summarized the available evidence from CTs that compared to the BLS group, the ALS group showed no significant improvement on in-hospital mortality, neurological outcomes, and time spent on scene in patients with trauma in the CTs design group. Moreover, the OS design groups showed increased mortality and time spent on scene in the ALS group.

Our results of CTs are consistent with the results of a previous meta-analysis which was reported in 2011 and ALS care was not associated with increased survival in trauma patients (32). The authors retrieved data from 9 trials including 16,857 patients that met their inclusion criteria (23, 25–27, 33–37) and included helicopter transportation. In the present study, we excluded helicopter transportation because resource was very limited, and it could affect results; tracheal intubation or chest compressions are difficult to perform in a flying helicopter. In the studies that met our criteria, the patients were mostly transported by ambulance and there was no difference in outcomes between the ALS and BLS groups.

We performed meta-analysis using observational studies to confirm the robustness of results. Regarding the OS design group, ALS prolonged time spent on scene and increased in-hospital mortality compared to BLS, although certainty of evidence was very low. A previous study showed that ALS was associated with an increased mortality rate compared to BLS (31). In this previous study, the time spent on scene was higher for ALS than for BLS providers (18.5 min vs. 13.5 min, *p* = 0.005) (31); this can affect mortality. Another observational study reported that an increase in total prehospital time was associated with increasing in-hospital mortality in trauma patients (38). These results are correlated with our OS design group results.

Our findings showed no ALS advantages in both CTs and OS groups. Previous studies regarding ALS procedures reported that endotracheal intubation in prehospital settings has not been shown to reduce mortality and morbidity in severe trauma patients. Moreover, performing ALS procedures in a difficult task under trying conditions and could be harmful (27, 39–41). Endotracheal intubation by unskilled practitioners could result in adverse events and result in low quality of chest compressions with significant interruptions (39). The value of prehospital IV fluid resuscitation has also been questioned (42–44). IV infusions of crystalloid may promote hemorrhage by diluting coagulation factors and by lowering blood viscosity (42). Theoretically, these previous reports of ALS procedures in prehospital settings may support our findings. However, caution is required to interpret our results. Recently, ALS equipment has improved; tracheal intubation using a video laryngoscope are being introduced. In the future, ALS may improve clinical outcomes due to advances in resuscitation equipment.

There are number of strengths in the present study. A major strength of this analysis is that the present study was evaluated by the quality of the evidence by the GRADE approach, which is widely accepted, and which offers an objective system for rating quality of evidence in systematic reviews and clinical practice guidelines. The other strength of this study is that could include both CTs and OS design. Our findings therefore become more robust.

The present study has several limitations. First, the meta-analyses were based on data from only two CTs and eight observational studies. Two CTs are not enough and observational studies have a limited ability to control for confounding variables, and the retrospective designs must be interpreted with particular attention. More severe patients might include in the ALS group than the BLS group in OS design. Thus, we judged the quality of the evidence provided by the observational studies as “very low.” Second, procedures of ALS or eligible populations were varied in the individual studies. Third, we could not perform subgroup analysis such as divide trauma into blunt and penetrating because of insufficient data from the included studies.

## Conclusion

The present study showed no advantage of prehospital ALS intervention on in-hospital mortality, neurological outcomes, and spending time on scene in prehospital trauma patients. Immediate definitive treatment may be important for trauma and should avoid prolonged time spent on scene. Further studies are warranted to validate our results.

## Data Availability Statement

Publicly available datasets were analyzed in this study. The data and material used for this meta-analysis are contained in our list of references.

## Author Contributions

YK conceived the idea for this systematic review and drafted the manuscript. YK, TF, RU, MK, SK, and HS developed the methodology. TF, RU, MK, SK, HS, TH, and KH revised the manuscript. All authors critically reviewed and approved the final manuscript.

## Conflict of Interest

The authors declare that the research was conducted in the absence of any commercial or financial relationships that could be construed as a potential conflict of interest.

## Abbreviations

ALS: advanced life support
BLS: basic life support
CRD: center for reviews and dissemination
CTs: controlled trials
CBAs: controlled before-and-after studies
RCTs: randomized controlled trials
OS: observational studies
OR: Odds ratios
CI: confidence intervals.
