# Supplementary material for: Advanced Life Support vs. Basic Life Support for Patients With Trauma in Prehospital Settings: A Systematic Review and Meta-Analysis
Source: Front Med (Lausanne). 2021 Mar 26;8:660367. doi: 10.3389/fmed.2021.660367 (PMC8032986; doi:10.3389/fmed.2021.660367)
Supplement: Supplementary file 1 [file Data_Sheet_1.PDF]

Additional file 1 Search strategies

Database	Date of search	Search terms
MEDLINE (source, PubMed), EMBASE, Cochrane Central Register of Controlled Trials	Until end of August, 2017	#1: “Trauma” OR “Injury” OR “Wound” OR OR “Heart Arrest”<br>#2: “Advanced Cardiac Life Support” OR “Basic Life Support” OR “Life Support Care”<br>#3: “prehospital” OR “Emergency Medical Services” OR “first Aid” OR “Resuscitation”<br><br>#4: #1 AND #2 AND #3, Filters: Humans: Adult (19+ years)
