# Supplementary material for: Advanced Life Support vs. Basic Life Support for Patients With Trauma in Prehospital Settings: A Systematic Review and Meta-Analysis
Source: Front Med (Lausanne). 2021 Mar 26;8:660367. doi: 10.3389/fmed.2021.660367 (PMC8032986; doi:10.3389/fmed.2021.660367)
Supplement: Supplementary file 2 [file Data_Sheet_2.PDF]

Additional file 2.

(A)

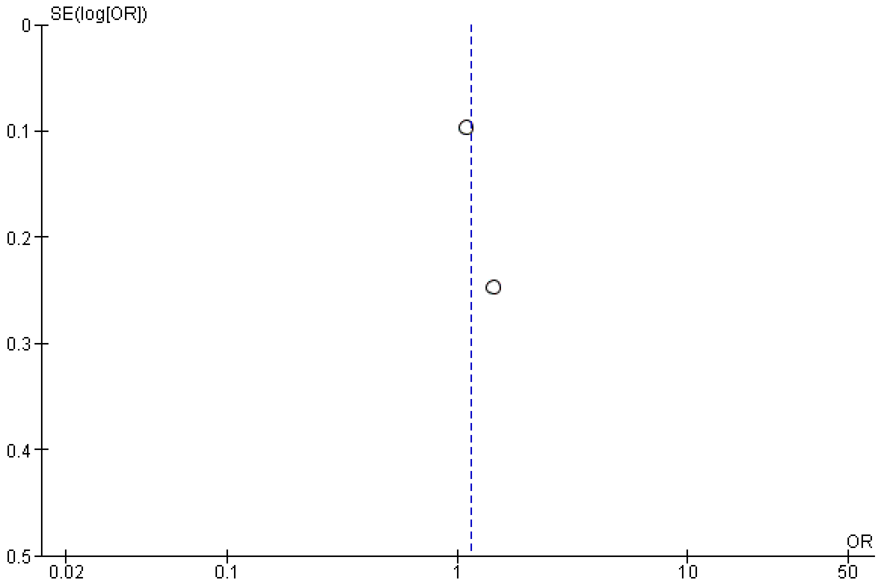

(B)

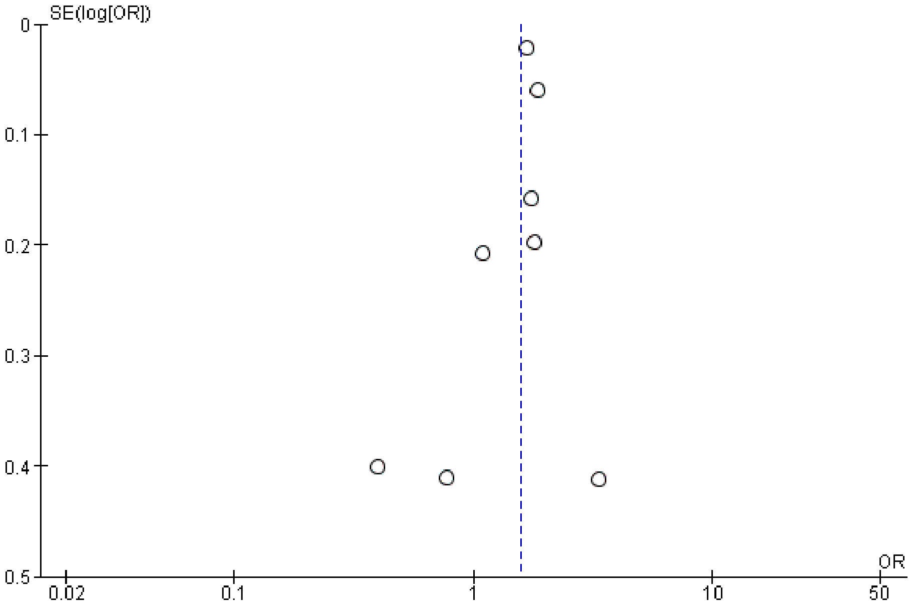

Funnel plots for controlled trials design (A) and observational studies design (B).
OR, odds ratio
